# Predictors of aetiology and outcomes of acute gastrointestinal illness in returning travellers: a retrospective cohort analysis

**DOI:** 10.1186/s12879-021-06223-3

**Published:** 2021-06-23

**Authors:** Robert A. Lever, Louis Tapper, Sophie Skarbek, Peter L. Chiodini, Margaret Armstrong, Robin L. Bailey

**Affiliations:** 1 Hospital for Tropical Diseases, Maple House, Tottenham Court Road, London, UK; 2 University College London Division of Infection and Immunity, London, UK; 3 University College London Medical School, London, UK; 4 London School of Hygiene and Tropical Medicine, London, UK

**Keywords:** Returning travellers, Diarrhoea, Parasitic disease, Gastrointestinal illness

## Abstract

**Background:**

Gastrointestinal illness is a major cause of morbidity in travellers and is a common reason for presentation to healthcare services on return. Whilst the aetiology of imported gastrointestinal disease is predominantly infectious, outcomes are variable due to a range of phenomena such as post-infectious irritable bowel syndrome, drug resistance and occult pathology (both infectious and non-infectious). Previous studies have focussed on predictors of aetiology of gastrointestinal disease in travellers; we present a retrospective study combining both aetiological and early outcome data in a large cohort of returned travellers.

**Method:**

We identified 1450 patients who attended our post-travel walk-in clinic with gastrointestinal symptoms between 2010 and 2016. Demographic, travel, clinical and laboratory data was collected through case note review. Logistic regression analysis to examine correlates of aetiology and outcome were performed in R (CRAN Project 2017).

**Results:**

Of 1450 patients in our cohort 153 reported bloody diarrhoea and 1081 (74.6%) reported non-bloody diarrhoea. A definitive microbiological diagnosis was made in 310 (20.8%) of which 137 (9.4%) had a parasite identified and 111 (7.7%) had a bacterial cause identified. Factors associated with a parasitological diagnosis included history of travel to South Asia (aOR = 2.55; 95%CI 1.75–3.70, *p* < 0.0001) and absence of bloody diarrhoea (aOR = 0.22; 95%CI 0.066–0.53, *p* < 0.005). Factors associated with a bacteriological diagnosis included male gender (aOR = 1.69; 95%CI 1.10–2.62, *p* < 0.05), an age < 37 years on presentation (aOR = 2.04; 95%CI 1.25–3.43, *p* < 0.01), white cells on stool microscopy (aOR = 3.52; 95%CI 2.09–5.86, *p* < 0.0001) and a C-reactive protein level of >5iu/dL (aOR = 4.68; 95%CI 2.91–7.72, *p* < 0.0001). The majority (1235/1450, 82.6%) reported full symptomatic resolution by the first follow up visit; factors associated with lack of symptomatic resolution included female gender (aOR = 1.45 95%CI 1.06–1.99, *p* < 0.05), dysenteric diarrhoea (aOR = 2.14 (95%CI 1.38–3.25, *p* < 0.0005) and elevated peripheral leukocyte count (aOR = 1.58 95%CI 1.02–2.40, *p* < 0.05).

**Conclusions:**

In a cohort of returned travellers, we were able to identify multiple factors that are correlated with both aetiology and outcome of imported gastrointestinal syndromes. We predict these data will be valuable in the development of diagnostic and therapeutic pathways for patients with imported gastrointestinal infections.

**Supplementary Information:**

The online version contains supplementary material available at 10.1186/s12879-021-06223-3.

## Key points

Gastrointestinal pathology is common in returned travellers. In this large retrospective study, we identify a number of demographic, clinical and laboratory features which are associated with the aetiology and clinical outcome of imported enteric diseases.

## Introduction

Diarrhoea and other gastrointestinal diseases are extremely common in travellers and remain a key cause of morbidity in this group despite reports of reducing incidence of foodborne infection worldwide [1–4]. Multiple studies including large-scale GeoSentinel analyses have estimated between 20 and 50% of travellers experience gastrointestinal symptoms related to travel; this risk is enhanced in lower- and middle-income countries with up to 40,000 travellers to these destinations experiencing symptoms per day [5–9]. Imported gastrointestinal disease represents a spectrum of different clinical syndromes of which acute diarrhoeal illness is the most common, accounting for 60% or more of all presentations to medical care on return [1, 5, 6].

The aetiology of imported gastrointestinal pathology is predominantly infectious in nature and microbiological identification of the causative agent is successful in between 20 and 94% of patients with acute diarrhoeal illness [1, 5, 10]. Most cases of imported diarrhoea are bacterial in origin [1]. However, in a large GeoSentinel study, in the 39% of returning travellers with any gastrointestinal syndrome who received a microbiological diagnosis, approximately twice as many had a parasite identified (65%) as those who had a bacterial cause isolated (31%) [6]. A previous report from our centre identified a bacterial origin for symptoms in 12.5% and a parasitic cause in 11.9% of patients with acute diarrhoea [11]. Bacterial causes of acute diarrhoea typically have a shorter incubation and duration than those of parasitic origin, and this may explain previous reported disparities in comparative aetiological prevalence [9, 11]. Identification of factors which predict aetiology of imported gastrointestinal disturbance are therefore of interest as they may help guide empirical therapy, prognosis and follow up [1, 9, 11] Limited work has previously been done in this arena; in this work we seek to extend and strengthen these earlier observations [11].

The majority of infective gastrointestinal disease, and particularly diarrhoeal illness, is short-lived and self-limiting, with an average duration of 4–5 days [12]. However, long term complications are well-recognised of which post-infectious irritable bowel syndrome (PI-IBS), characterised by a persistence in gastrointestinal distress after convalescence, is best described and occurs in nearly 1 in 5 patients [13, 14]. Persistence of symptoms after less common imported gastrointestinal syndromes such as isolated abdominal pain or bloating are less well described. Precipitators of non-resolution of symptoms after treatment for an acute imported gastrointestinal infection can be broadly divided into five categories: resistance of pathogen to empirical treatment, failure of host response (e.g. immunocompromise), cryptic infection, primary non-infectious pathology (such as undiagnosed inflammatory bowel disease) and, functional post-infectious bowel abnormalities, of which PI-IBS is the most well described [13–19]. As functional bowel disease is a diagnosis of exclusion, the initial evaluation of patients with recalcitrant gastrointestinal symptoms usually necessitates further laboratory and imaging investigations and may include invasive assessments such as endoscopy [1].

Early identification and effective treatment of travellers with persistent gastrointestinal symptoms is clinically challenging. Despite this, only a limited number of studies have directly looked at predictive factors for non-resolution of symptoms; we seek to address this in our study.

## Methods

### Clinical setting

The Hospital for Tropical Diseases (London, UK) operates a Walk-in Emergency clinic for any patient with symptoms following return from abroad. Patients self-refer and do not need a prior appointment or review by their primary healthcare practitioner before review. In order to be eligible for review patients must have returned from travel within the preceding 6 months. The clinic target population is adult travellers however older adolescents > 16 years are occasionally reviewed at clinician discretion. Other than this there are no pre-requisites for review. Each patient is assessed by a triage nurse and subsequently by a doctor, where an initial diagnosis is made, and emergency treatment is provided. A subset of these patients will return to clinic either as a planned follow-up or re-present due to symptom persistence. Patients routinely receive a call from a clinician within 7 days with the results of investigations performed at the time of review, at this time a brief update on symptoms is sought by the telephoning clinician; this process is independent of otherwise planned follow-up.

### Cohort selection

All patients with gastrointestinal symptoms presenting to the Hospital for Tropical Diseases Emergency Walk-in Clinic (London, UK) have a stool sample requested for analysis for ova, cysts and parasites (OCP) at triage. We identified stool samples submitted for stool OCP to the Hospital for Tropical Diseases Parasitology Department between January 2010 and January 2016. From this we identified patient-episodes corresponding to individual attendances at the clinic. Patients were deemed ineligible for analysis if they provided samples for asymptomatic screening for parasites in the context of another, non-gastrointestinal, illness and if the sample was derived from the parallel tertiary referral outpatient clinic which operates on the same site. This is due to the fact that other clinics operating on the same site do not restrict their patient population to recently returned travellers.

### Syndromic categorisation and outcome parameters

**Fig. 1:**
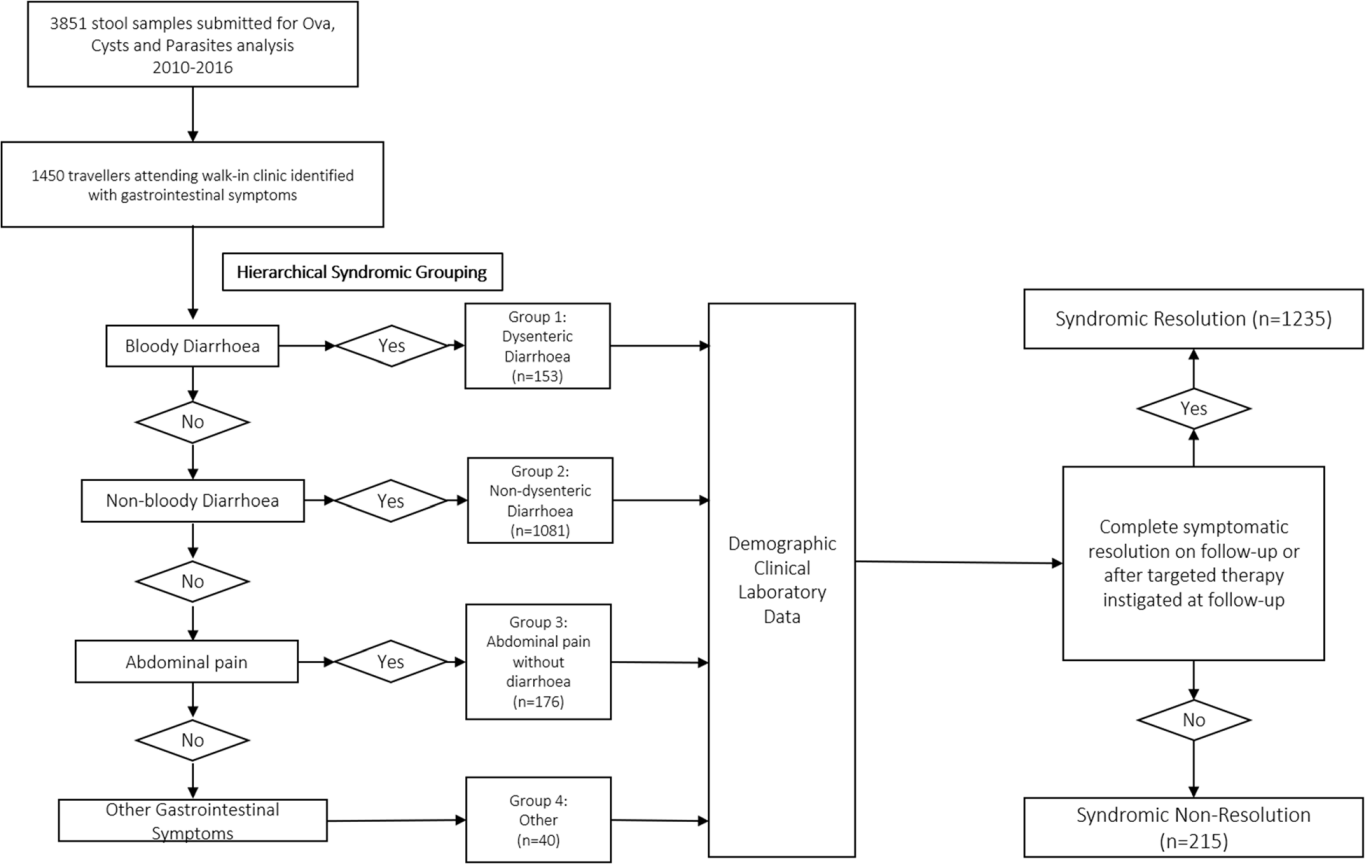
Cohort Identification Methodology and Hierarchical Syndromic Grouping

Patients were grouped into syndromic categories as described in Fig. 1. Presence of the first indicator symptom as detailed in the flow chart led to inclusion in that category irrespective of other symptoms. This allowed delineation of potentially overlapping gastrointestinal symptom clusters; for example, a patient with both dysenteric diarrhoea and abdominal pain would be categorised according to the higher ranked symptom (dysenteric diarrhoea) for the purposes of syndrome grouping for analysis.

The primary assessed outcome was complete resolution of symptoms. This did not require only the resolution of the highest graded symptom, but the absence of any significant symptoms as reported by the patient. For example, a patient in Group 1 (dysenteric diarrhoea) at first presentation would be categorised as “Syndromic non-resolution” if they had persistent non-bloody diarrhoea at the time of review.

### Data collection

Routine data were collected via audit of historical clinical records by clinical staff and anonymised before entry onto a database. Scope of data included demographic details, clinical data from correspondence and laboratory data from electronic records. All data were collected in compliance with locally established audit standards and personal data were anonymised in compliance with GDPR legislation (European Union 2018).

### Ethics and governance

All methods and protocols employed within this study were approved by the Hospital for Tropical Diseases Audit Committee (London, UK) in accordance with legislation and regulations laid out by the NHS Human Research Authority (UK).

### Data analysis

Data were analysed in Microsoft Excel (Microsoft Corporation 2018) and R (R Development Core Team, 2008). Students’ t-test was used for normally distributed continuous variables, 2 by 2 tables were analysed using Chi squared or Fisher’s Exact Test. Logistic regression modelling was performed with binomial distribution parameters. Adjustment was made for major confounders including age and gender and suspected interacting variables identified through univariate analysis. Further details and code used for analysis may be found on github.com/rlever/HTDgastro. Maps were produced with the MS Excel 3D Maps plugin and rworldmaps package for R (CRAN 2017).

## Results

### Cohort characteristics

**Fig. 2:**
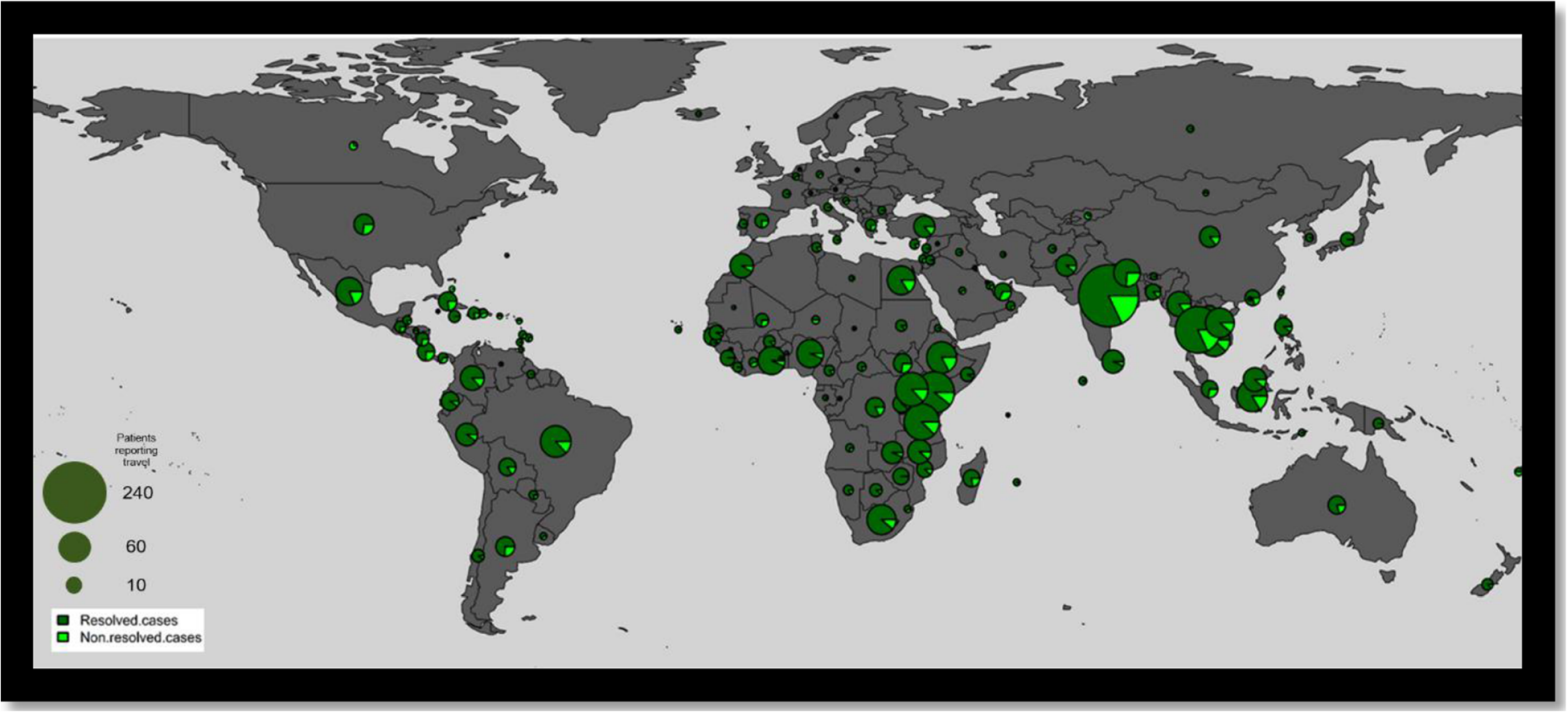
Patient Travel Destination Circle size indicates number of patients as referenced in left-hand scale. Light green segments indicate proportion of patients without syndromic resolution at follow-up

From 3851 stool samples submitted for stool OCP analysis, we identified 1450 consecutive patients who had attended the Emergency Walk-in clinic between January 2010 and January 2016 who had a primary gastrointestinal syndrome after return from abroad. 819 patients (56.5%) were female, and the mean age was 35.97 years (IQR 27.3–42.4 years). 445 (30.1%) of reviewed patients had visited more than one country during their trip and 430 (29.7%) had visited more than one geographical region of the world. The top geographical regions visited were South East Asia (449/1450 31.0%), South Asia (356/1450 24.6%) and East Africa (323/1450 22.3%). (Table 1.; Fig. 2.)

**Table 1:** Clinical Characteristics – Parasitic and Bacterial Diagnosis

	Any Parasitic Diagnosis (***N*** = 137)	***p*** value	Any Bacterial Diagnosis (***N*** = 111)	***p*** value	Total (***N*** = 1450)
**Gender**		0.802		*0.006	
Female	76 (55.5%)		49 (44.1%)		819 (56.5%)
Male	61 (44.5%)		62 (55.9%)		631 (43.5%)
**Age**		0.276		*0.018	
Mean (SD)	37.065 (12.685)		33.302 (11.987)		35.971 (12.353)
Range	16.720 - 73.200		16.930 - 73.200		15.240 - 84.540
**HIV Positive**	0 (0.0%)	0.901	0 (0.0%)	0.811	2 (0.1%)
**Travel History**					
Central Asia	6 (4.4%)	0.176	2 (1.8%)	0.574	38 (2.6%)
Europe	3 (2.2%)	0.337	2 (1.8%)	0.279	53 (3.7%)
North Africa	11 (8.0%)	0.151	14 (12.6%)	0.781	171 (11.8%)
Pacific Islands	0 (0.0%)	0.518	1 (0.9%)	0.191	4 (0.3%)
Southern Africa	5 (3.6%)	0.165	2 (1.8%)	*0.039	93 (6.4%)
Caribbean	1 (0.7%)	0.038	4 (3.6%)	0.796	59 (4.1%)
South America	14 (10.2%)	0.484	8 (7.2%)	0.581	125 (8.6%)
Australia and New Zealand	2 (1.5%)	0.901	2 (1.8%)	0.85	23 (1.6%)
Bahamas	0 (0.0%)	0.575	0 (0.0%)	0.618	3 (0.2%)
Middle East	1 (0.7%)	0.12	1 (0.9%)	0.203	41 (2.8%)
South Asia	54 (39.4%)	***< 0.001***	14 (12.6%)	*0.013	319 (22.0%)
Central America	8 (5.8%)	0.676	6 (5.4%)	0.573	97 (6.7%)
West Africa	13 (9.5%)	0.527	7 (6.3%)	0.094	161 (11.1%)
South East Asia	24 (17.5%)	0.433	31 (27.9%)	*0.031	291 (20.1%)
East Africa	21 (15.3%)	0.404	26 (23.4%)	0.116	260 (17.9%)
North America and Canada	1 (0.7%)	0.264	3 (2.7%)	0.582	29 (2.0%)
Central Africa	3 (2.2%)	0.766	1 (0.9%)	0.436	27 (1.9%)
China	1 (0.7%)	0.145	2 (1.8%)	0.574	38 (2.6%)
Oceania	0 (0.0%)	0.428	1 (0.9%)	0.405	6 (0.4%)
**Syndrome**		*0.008		***< 0.001***	
Non-dysenteric diarrhoea	117 (85.4%)		81 (73.0%)		1081 (74.6%)
Abdominal pain	13 (9.5%)		3 (2.7%)		176 (12.1%)
Dysenteric diarrhoea	4 (2.9%)		25 (22.5%)		153 (10.6%)
Other	3 (2.2%)		2 (1.8%)		40 (2.8%)
**Stool Microscopy**					
White cells	13 (9.5%)	0.751	36 (32.4%)	***< 0.001***	127 (8.8%)
Red cells	8 (5.8%)	0.342	18 (16.2%)	***< 0.001***	62 (4.3%)
**Peripheral WBC Count**		0.066		0.334	
Decreased	1 (0.7%)		0 (0.0%)		14 (1.0%)
Increased	23 (16.8%)		15 (13.5%)		159 (11.0%)
Normal	110 (80.3%)		88 (79.3%)		1206 (83.2%)
**C-reactive Protein**		0.151		***< 0.001***	
Increased	44 (32.1%)		77 (69.4%)		500 (34.5%)
Normal	90 (65.7%)		26 (23.4%)		873 (60.2%)
**ALT**		0.134		0.588	
Increased	25 (18.2%)		14 (12.6%)		197 (13.6%)
Normal	108 (78.8%)		89 (80.2%)		1178 (81.2%)

Significance indicated by *p* value marked in bold with * where ≤0.001 < *p* < 0.05

### Syndromic presentations of gastrointestinal disease

Imported gastrointestinal disease encompasses a spectrum of clinical presentations; to capture this in a clinician applicable manner we separated patients into 4 syndromic categories. Group 1: Dysenteric diarrhoea (defined as a diarrhoeal illness with the presence of blood in the stool); Group 2: Non-dysenteric diarrhoea (defined as any diarrhoeal illness without the presence of blood); Group 3: Abdominal pain/Bloating (defined as the presence of abdominal pain and/or bloating without diarrhoea); Group 4: Other (defined as all other gastrointestinal syndromes not captured in Groups 1–3). Group 4 included those who reported isolated nausea or vomiting, passage of a suspected helminth in stool, suspected helminth in emesis, altered bowel habit without diarrhoea, pruritis ani, constipation, and oral ulceration.

The commonest syndrome in our cohort was Non-dysenteric diarrhoea (1081 patients, 74.6%) followed by Abdominal pain/Bloating (176 patients, 12.1%) and Dysenteric diarrhoea (153 patients, 10.6%) (Fig. 1.).

### Laboratory investigation of patients presenting to the walk-in clinic

All our patients had microscopy performed on a stool concentrate for ova, cysts and parasites. In addition, 90.7% of patients underwent bacterial stool culture and 42.1% had molecular analysis for *Entamoeba histolytica*, *Giardia intestinalis* and *Cryptosporidium* performed via multiplex polymerase chain reaction. Peripheral blood sampling was performed in the majority of patients – 95.1% had full blood count (FBC), 94.7% had C-reactive protein and 94.8% had liver function tests performed respectively. 43.8% underwent testing for HIV infection.

### Aetiology of gastrointestinal disease in returning Travellers

301 patients (20.8%) received a definitive diagnosis as a result of their interaction with our travellers’ clinic of which 242 (80.3%) were as a result of microbiological and parasitological analysis of stool.

The presence of a parasite was confirmed in 137 patients (9.4%) and a bacterial pathogen was identified in 111 patients (7.7%). The commonest identified gastrointestinal pathogen in our cohort was *Giardia intestinalis* which was identified in 92 patients (6.3%). The commonest causes of bacterial gut infection were *Salmonella* spp. (39 cases, 2.7%) and *Campylobacter* spp. (47 cases, 3.2%).

Patients in whom a parasite was identified were more likely to fall into syndromic Group 2 (non-dysenteric diarrhoea) (Table 1). Of note 6 of 7 *E.*histolytica infections identified by PCR presented with non-dysenteric diarrhoea. Reported travel to South Asia was associated with an increased risk for detection of a parasite during clinical workup (aOR = 2.55; 95%CI 1.75–3.70, *p* < 0.0001) and particularly for *Giardia intestinalis* infection (aOR = 3.18; 95% CI = 2.05–4.92, *p* < 0.00001); correspondingly, those who reported dysenteric diarrhoea were significantly less likely to have a parasite identified during testing (aOR = 0.22; 95%CI 0.066–0.53, *p* < 0.005) (Table 2).

**Table 2:** Predictors of Aetiology of Imported Gastrointestinal Disease

	Resolution (***N*** = 1235)	Non-resolution (***N*** = 215)	Total (***N*** = 1450)	***p*** value
**Gender**				***0.043***
Female	684 (55.4%)	135 (62.8%)	819 (56.5%)	
Male	551 (44.6%)	80 (37.2%)	631 (43.5%)	
**Age**				0.914
Mean (SD)	35.986 (12.241)	35.888 (13.009)	35.971 (12.353)	
Range	16.000 - 84.540	15.240 - 78.340	15.240 - 84.540	
**Travel History**				
Central Asia	31 (2.5%)	7 (3.3%)	38 (2.6%)	0.528
Europe	46 (3.7%)	7 (3.3%)	53 (3.7%)	0.735
North Africa	144 (11.7%)	27 (12.6%)	171 (11.8%)	0.706
Pacific Islands	2 (0.2%)	2 (0.9%)	4 (0.3%)	***0.047***
Southern Africa	84 (6.8%)	9 (4.2%)	93 (6.4%)	0.149
Caribbean	44 (3.6%)	15 (7.0%)	59 (4.1%)	***0.019***
South America	108 (8.7%)	17 (7.9%)	125 (8.6%)	0.686
Bahamas	1 (0.1%)	2 (0.9%)	3 (0.2%)	***0.011***
Middle East	32 (2.6%)	9 (4.2%)	41 (2.8%)	0.193
Australia and New Zealand	19 (1.5%)	4 (1.9%)	23 (1.6%)	0.727
South Asia	264 (21.4%)	55 (25.6%)	319 (22.0%)	0.17
Central America	79 (6.4%)	18 (8.4%)	97 (6.7%)	0.285
West Africa	144 (11.7%)	17 (7.9%)	161 (11.1%)	0.106
South East Asia	248 (20.1%)	43 (20.0%)	291 (20.1%)	0.978
East Africa	227 (18.4%)	33 (15.3%)	260 (17.9%)	0.285
North America and Canada	19 (1.5%)	10 (4.7%)	29 (2.0%)	***0.003***
China	32 (2.6%)	6 (2.8%)	38 (2.6%)	0.866
Oceania	6 (0.5%)	0 (0.0%)	6 (0.4%)	0.306
Central.Africa	22 (1.8%)	5 (2.3%)	27 (1.9%)	0.586
**Syndrome**				***0.002***
Non-dysenteric diarrhoea	936 (75.8%)	145 (67.4%)	1081 (74.6%)	
Abdominal pain	146 (11.8%)	30 (14.0%)	176 (12.1%)	
Dysenteric diarrhoea	116 (9.4%)	37 (17.2%)	153 (10.6%)	
Other	37 (3.0%)	3 (1.4%)	40 (2.8%)	
**Stool Microscopy**				
White cells	108 (8.7%)	19 (8.8%)	127 (8.8%)	0.965
Red cells	51 (4.1%)	11 (5.1%)	62 (4.3%)	0.509
**Peripheral WBC Count**				0.251
Decreased	12 (1.0%)	2 (0.9%)	14 (1.0%)	
Increased	127 (10.3%)	32 (14.9%)	159 (11.0%)	
Normal	1034 (83.7%)	172 (80.0%)	1206 (83.2%)	
**C-reactive Protein**				0.209
Increased	435 (35.2%)	65 (30.2%)	500 (34.5%)	
Normal	732 (59.3%)	141 (65.6%)	873 (60.2%)	
**ALT**				0.447
Increased	164 (13.3%)	33 (15.3%)	197 (13.6%)	
Normal	1004 (81.3%)	174 (80.9%)	1178 (81.2%)	
**Any Definitive Diagnosis**	261 (21.1%)	40 (18.6%)	301 (20.8%)	0.399
**Microbiological Diagnosis**	214 (17.3%)	28 (13.0%)	242 (16.7%)	0.153
Bacterial	100 (8.1%)	11 (5.1%)	111 (7.7%)	0.118
Parasitic	119 (9.6%)	18 (8.4%)	137 (9.4%)	0.559
**Diagnosis**				
*Giardia intestinalis*	78 (6.3%)	14 (6.5%)	92 (6.3%)	0.913
*Entamoeba histolytica*	6 (0.5%)	1 (0.5%)	7 (0.5%)	0.968
*Blastocystis hominis*	27 (2.2%)	2 (0.9%)	29 (2.0%)	0.225
*Cryptosporidium parvum*	13 (1.1%)	2 (0.9%)	15 (1.0%)	0.87
*Cyclospora cayatanensis*	6 (0.5%)	0 (0.0%)	6 (0.4%)	0.306
*Campylobacter spp.*	43 (3.5%)	4 (1.9%)	47 (3.2%)	0.215
*Shigella spp.*	22 (1.8%)	3 (1.4%)	25 (1.7%)	0.688
*Salmonella spp.*	35 (2.8%)	4 (1.9%)	39 (2.7%)	0.415
*Plesiomonas shigelloides*	5 (0.4%)	0 (0.0%)	5 (0.3%)	0.35

Patients with a proven bacterial origin to their symptoms had a younger mean age (33.3 vs 36.0 years, *p* = 0.016) and were more likely to fall into syndromic Group 1 (dysenteric diarrhoea) (Table 1) After adjustment for confounders male gender was significantly associated with a confirmed bacterial aetiology (aOR = 1.69; 95%CI 1.10–2.62, *p* < 0.05), an age < 37 years on presentation (aOR = 2.04; 95%CI 1.25–3.43, *p* < 0.01), presence of white cells on stool microscopy (aOR = 3.52; 95%CI 2.09–5.86, *p* < 0.0001) and a C-reactive protein level of >5iu/dL (aOR = 4.68; 95%CI 2.91–7.72, *p* < 0.0001) (Table 2). These data are consistent with previously published observations from our unit [11].

### Outcomes of gastrointestinal disease in returning Travellers

Persistent abdominal symptoms are a common feature of returning travellers suffering from gastrointestinal pathology. To assess the prevalence of persistent non-resolution of symptoms within our cohort we identified the patients who had any ongoing symptoms, either at follow up after empirical treatment or the first follow up after a specific identified aetiology was identified. Those who failed to attend a pre-arranged follow up appointment were assumed to have syndromic resolution.

Of 1450 returning travellers, 215 (17.4%) had non-resolution of their symptoms at follow up; the comparative travel histories are shown in Fig. 2. A higher proportion of patients with persistent symptoms compared to those with complete resolution were female (62.8% vs 55.4%) and were more likely to have travelled to the Caribbean, Pacific Islands, Bahamas and North America respectively in a univariate analysis (Table 3). Dysenteric diarrhoea as a presenting syndrome was over-represented in those with persistent symptoms at follow up (17.2% vs 9.4% of cases) however the presence of red or white blood cells on stool microscopy was not significantly different between the two groups (Table 3). No individual microbiological or parasitological diagnosis was associated with non-resolution of symptoms (Table 3). These findings may be related to new presentations of non-travel related pathology such as inflammatory bowel disease in these patients as has been previously described by our centre [20].

**Table 3:** Clinical Characteristics – Outcome at first follow-up

	**Odds Ratio for Parasitic Diagnosis**	**95% CI**	***p*** **value**
Male Gender	1.07	0.74-1.53	0.721
Age >37 years	1.42	0.98-2.04	0.058
**Travel to South Asia**	**2.55**	**1.75-3.70**	**<0.0001**
Syndrome - Abdominal Pain and Bloating	0.68	0.36-1.20	0.214
**Syndrome - Dysenteric Diarrhoea**	**0.22**	**0.066-0.53**	**<0.005**
Syndrome - Other GI syndrome	0.70	0.16-2.00	0.556
	**Odds Ratio for Bacterial Diagnosis**	**95% CI**	***p*** **value**
**Male Gender**	**1.69**	**1.10-2.62**	**<0.05**
**Age <37 Years**	**2.04**	**1.25-3.43**	**<0.01**
**Travel to South Asia**	**0.47**	**0.24-0.83**	**<0.013**
**Travel to Southern Africa**	**0.11**	**0.006-0.52**	**<0.05**
**Stool Microscopy - White cells**	**3.52**	**2.09-5.86**	**<0.0001**
**CRP >5iu/dL**^a^	**4.68**	**2.91-7.72**	**<0.0001**

^a^Cases where CRP not performed removed from analysis

In a multivariate analysis female gender was associated with an hazard ratio of 1.45 (95%CI 1.06–1.99, *p* < 0.05) for persistence of symptoms in our cohort (Table 4). An initial presenting complaint of dysenteric diarrhoea, and those with a measured peripheral leucocytosis at presentation were associated with an hazard ratio of 2.14 (95%CI 1.38–3.25, *p* < 0.0005) and 1.58 (95%CI 1.02–2.40, *p* < 0.05) respectively for non-resolution of symptoms (Table 4). Additionally, after adjustment for other factors, travel to North America (USA and Canada) was significantly associated with ongoing symptoms at follow-up (HR 3.61, 95%CI 1.57–7.9, *p* < 0.005) (Table 4).

**Table 4:** Predictors of Outcome of Imported Gastrointestinal Syndromes

	Odds Ratio for Persistence of Symptoms	95% CI	Pr(>|z|)
**Female gender**	**1.45**	**1.06-1.99**	**<0.05**
Age - 28-37 years	0.76	0.52-1.10	0.150
Age - >37 years	0.84	0.58-1.22	0.369
**Travel to North America**	**3.61**	**1.57-7.9**	**<0.005**
Syndrome - Abdominal Pain and Bloating	1.41	0.89-2.18	0.133
**Syndrome - Dysenteric Diarrhoea**	**2.14**	**1.38-3.25**	**<0.0005**
Syndrome - Other GI syndrome	0.58	0.14-1.67	0.380
**Leucocytosis**^a^	**1.58**	**1.02-2.40**	**<0.05**

^a^Cases where peripheral white cell count not performed removed from analysis

## Discussion

To our knowledge this is the largest contemporary study which focusses both on the aetiology and the outcomes of returning travellers with gastrointestinal symptoms. The results of this work therefore provide valuable data to inform both empirical treatment of imported gastrointestinal disease and facilitate the early identification of those patients who may have recalcitrant symptoms possibly due to non-infective causes and require follow-up.

Consistent with previous reports, our study demonstrates that despite extensive investigation, only a minority of patients with imported gastrointestinal disturbance receive a microbiological diagnosis but that the majority resolve completely with conservative, empirical, or targeted management.

In agreement with a smaller earlier report from our unit, travel to South Asia was associated with a positive parasitological diagnosis, of which infection with *Giardia intestinalis* was by far the most common. Similarly, the identification of a causative bacterial agent was associated with dysenteric symptoms, white cells on stool microscopy and an elevated C-reactive protein level, in accordance with the existing literature. Interestingly younger age and male gender were significantly associated with a positive bacterial culture. This may represent a true higher population prevalence in young male patients to imported gastrointestinal bacterial infection or may be related to a higher bacillary load in this population. This may suggest that a lower threshold for providing empiric antimicrobial therapy in this group may be advantageous depending on setting; this also highlights that this group may be worthy of further study in prospective therapeutic trials.

Persistent abdominal symptoms are recognised complications of travel related gastrointestinal disease and management of these presentations may be challenging. In our study a variety of demographic, travel, syndromic and laboratory factors were found to influence the persistence of symptoms at follow-up. Dysenteric diarrhoea, peripheral leucocytosis at presentation and female sex all predicted lack of resolution in our cohort. A travel history which included travel to North America was predictive of lack of resolution in this study; the most plausible explanation for this is a higher comparative prevalence of non-infectious compared to infectious aetiologies of gastrointestinal disease in high income settings. Non-infectious gastrointestinal disease is known to have a typically longer symptomatic course [21].

The strength of this study is that it may allow earlier identification of those who would benefit most from further investigations, such as abdominal imaging, endoscopy, and specialist blood tests at the point of presentation to healthcare providers upon return from abroad. Practically these data suggest that patients with dysenteric diarrhoea and a peripheral leucocytosis at assessment should be prioritised for active follow up in travel healthcare settings.

The retrospective nature of this study represents its key limitation. Unfortunately, this means that the pathways for investigation and follow-up were not consistent across all the cases included and led to our making several assumptions regarding resolution. Key amongst these was including those without follow-up data as resolution which risks biasing our cohort of non-resolving patients and highlighting confounders rather than true predictors of non-resolution. However, we believe this is significantly mitigated by the fact that all patients had a planned clinician telephone call within a week to inform patients of results which should have acted as a “safety net” in these cases to minimise those who had ongoing symptoms but no ongoing input from our service. In addition to this, the perceived and actual barriers to care in our setting were minimal as our clinic is free of charge and open to all who meet travel criteria. Finally, our cohort have already demonstrated a high degree of healthcare seeking behaviour in the context of their decision to self-refer to the clinic in the first instance. These facts notwithstanding, we believe it is still important to highlight this as a potential source of bias within our data and should be considered in their interpretation. Also, due to the breadth of our dataset and the complexity of assessing of potentially multiple separate travel histories, our dataset does not include robust data for time from travel to onset of symptoms or duration of symptoms. The link between shorter incubation period and bacterial aetiology of imported diarrhoea is well established and we hope that despite this limitation we have potentially identified a number of other predictive factors with respect to aetiology and outcome [21]. Similarly, our dataset is limited by our intentional omission of therapeutic data in our data collection strategy. These data were not collected as we felt the complexity and diversity of antimicrobial strategies which we would find in our retrospective cohort of patients would preclude any meaningful analysis as our group was intentionally broad and the question of individual therapeutic efficacy is not best addressed in a study such as this.

A further limitation of our study design was our classification of endpoints. To maximise our capture of ongoing symptoms in a complex syndromic population we broadened our definition of non-resolution in our cohort. Practically, this means that “Non-resolution” encompassed a variety of entities including partial symptomatic resolution, symptomatic deterioration, and relapse. All of these are recognised clinical outcomes in the context of imported gastrointestinal disease. By grouping of these different outcomes, we were able to broaden our search for predictors of non-improvement however this meant sacrificing resolution of patient level symptom timelines. Further delineation of this would best be performed in the context of a prospective study with fixed timepoint symptomatic assessments, our dataset did not allow such analysis. Additionally, we made the decision to not restrict non-resolution to the original group defining symptom (i.e. those with dysenteric diarrhoea would be classified as non-resolution in the presence of ongoing non-dysenteric diarrhoea at follow up). This was intentional as a major non-infectious differential diagnosis of persistent diarrhoea is inflammatory bowel disease and we therefore wished to capture these patients in the “non-resolving” group.

In conclusion, we have demonstrated a constellation of factors which may predict both the aetiology and prognosis of gastrointestinal disease in returning travellers. We hope this will aid clinicians with initial assessment of such patients and allow practical early triage of patients for enhanced follow up.

## Supplementary Information

**Additional file 1.**

## Data Availability

Primary code for analysis will be made available on http://www.github.com (@rlever). Further data sharing will be considered in accordance with NHS HRA regulations and accepted practice.
