# Supplementary material for: Predictors of aetiology and outcomes of acute gastrointestinal illness in returning travellers: a retrospective cohort analysis
Source: BMC Infect Dis. 2021 Jun 23;21:599. doi: 10.1186/s12879-021-06223-3 (PMC8220688; doi:10.1186/s12879-021-06223-3)
Supplement: Supplementary file 1 — Additional file 1. [file 12879_2021_6223_MOESM1_ESM.pdf]

Symptoms Reported in Group 4:Other (n=40)
Pruritis ani
Passage of suspected parasite/helminth in stool
Vomiting
Constipation
Altered bowel habit
Nausea
Presence of suspected worm in emesis
Oral ulceration

Supplementary Table 1
